# Mandibular advancement devices in obstructive sleep apnea: an updated review

**DOI:** 10.5935/1984-0063.20210032

**Published:** 2022

**Authors:** Izabella Paola Manetta, Dominik Ettlin, Pedro Mayoral Sanz, Isabel Rocha, Miguel Meira e Cruz

**Affiliations:** 1 Department of Otolaryngology Pontificia, Universidade Católica de Campinas, - Campinas - São Paulo - Brazil.; 2 University of Zurich, Center of Dental Medicine - Zurich - Zurich - Switzerland.; 3 Department of Reconstructive Dentistry and Gerodontology, School of Dental Medicine, University of Berne, Berne, Switzerland.; 4 Catholic University of Murcia, Dental Sleep Medicine - Murcia - Murcia - Spain.; 5 Cardiovascular Autonomic Function Lab, Centro Cardiovascular da Universidade de Lisboa, Lisbon School of Medicine, Lisbon, Portugal.; 6 Sleep Unit, Centro Cardiovascular da Universidade de Lisboa, Lisbon School of Medicine, Lisbon, Portugal.; 7 Neuroimune Pain Interface Lab, Faculdade São Leopoldo Mandic, Campinas, São Paulo, Brazil.; 8 International Center of Clinical Sleep Medicine and Research, Escola Bahiana de Medicina e Saúde Pública, Salvador - Bahia, Brazil.

**Keywords:** Sleep Apnea, Obstructive, Mandibular Advancement, Occlusal Splints

## Abstract

Obstructive sleep apnea (OSA) is the most prevalent sleep-disordered breathing in the adult population and if untreated remains a significant cause of morbidity and mortality. Continuous positive airway pressure (CPAP) therapy is still the gold standard treatment for OSA, but patient acceptance and adherence are often poor due to a multitude of factors, thereby compromising treatment success. Mandibular advancement devices (MADs) have been proposed not only as a first line therapy for symptomatic snoring patients, but also for those suﬀering from mild to moderate OSA, or those who refuse or do not tolerate CPAP. Yet, improved understanding of MAD regarding design, construction, and mechanisms of action is an important requirement to successfully implement MAD as a therapeutic tool. Therefore, the main focus of this paper is to focus on the general concepts and mechanisms of action of MAD, while highlighting important characteristics in the context of their use as a viable and effective treatment option for OSA patients.

## INTRODUCTION

Obstructive sleep apnea (OSA), the most prevalent clinical entity in the spectrum of sleep-disordered breathing among adults and children, is defined by the presence of repeated episodes of complete or partial obstruction of the upper airways during sleep, despite ongoing respiratory efforts^1^. Its high prevalence, currently estimated as affecting nearly 1 billion people worldwide, imposes significant morbidity and mortality, such that OSA has clearly emerged as a major public health issue, while leading to major financial and social burden on both healthcare systems and society in general^2^.

The daytime consequences of OSA include a variety of symptoms, the most insidious being excessive daytime sleepiness (EDS), neurocognitive and behavioral impairments, and mood disorders^3^. In addition, OSA has clearly emerged as an independent risk factor for cardiovascular and metabolic diseases, such as hypertension, coronary artery disease, and endothelial dysfunction leading to myocardial ischemia, cardiac arrhythmias, stroke, as well as promoting or exacerbating dyslipidemias, insulin resistance, and type 2 diabetes mellitus^4-12^.

The pathophysiology of OSA is complex, involving a multitude of genetic, craniofacial - anatomical, neuromuscular, and inflammatory factors whose contributions differ from patient to patient^13^. Furthermore, OSA and insomnia may frequently co-occur challenging the usual standards of care^14^. In an effort to preserve the patency of the upper airway during sleep, various treatment modalities have been proposed^13^. Continuous positive airway pressure therapy (CPAP), initially developed by Sullivan et al.^15^, in the early 1980s, is still considered the most efficient therapeutic approach for OSA, since it stents the upper airway open throughout the respiratory cycle. Accordingly, CPAP has not only established itself as the gold standard and first line of treatment but has further shown its efficacy in a multitude of trials in which improvements in EDS, quality of life, and reductions in systemic blood pressure have clearly emerged^16^. Despite these benefits, patient acceptance, and adherence of CPAP is often poor, with attendant consequences^17^.

### Oral sleep medicine field and mandibular advancement devices

Oral sleep medicine (OSM) is an area of dental medicine that investigates and diagnoses sleep-disordered breathing (SDB) and its oral and maxillofacial determinants, while also exploring its consequences on general health and sleep^18^. Increased recognition of SDB has resulted in major expansion of maxillofacial surgical techniques and approaches aimed at correcting the underlying respiratory disturbance during sleep. In parallel, OSM has led to the development and to an incremental use of oral appliances (OA)^19,20^. Among the many types of OA, mandibular advancement devices (MAD), and tongue retainers (TR), have been incorporated as the most commonly used types of intraoral devices. These types of appliances underwent substantial developments over time and have been adopted as a standard therapeutic option in the clinical practice of sleep medicine. However, TR have not been extensively evaluated, are considered less effective than MAD and have been associated with lower patient adherence when compared to MAD^19-32^. Notwithstanding, TR are still indicated for partial or total edentulous patients who use removable prosthetic dentures, which restrict MAD use, and may be also of benefit among those patients who have limited mandibular protrusion, with a large tongue, or those suffering from acute temporomandibular disorders, or from advanced periodontal disease^21,22,31,33^.

The primary focus of this paper is to discuss the general concepts and mechanisms of action that govern development and application of MAD as a treatment option for patients suffering from OSA.

### MAD: concepts and mechanisms of action

MAD developed for treatment of sleep-disordered breathing are devices used in the oral cavity during sleep with the purpose of preventing the collapse between oropharyngeal tissues and the base of the tongue^21^. In general, MAD are geared to generate mandibular advancement and stabilization during sleep^19^ by promoting anterior traction displacement of the mandible with subsequent increases in the tension of the genioglossus muscle and the supra and infrahyoid muscles, expanding the air space in the pharyngeal region^22^.

A recent study evaluated skeletal/dental changes in a three-dimensional form using cone beam tomography during the use of MAD. It has been shown that mandibular protrusion promotes a linear vertical increase between the mandible and the maxilla and an anterosuperior displacement and rotation of the hyoid bone. Both of these features can assist professionals in deciding on the best candidates for this type of treatment^23^. Treatment with MAD aims to maintain the upper airways open during sleep by decreasing its resistance as well as the frequency and/or duration of the apneas and hypopneas, arousals related to respiratory effort, and snoring events^24^. In addition, MAD improve nighttime oxygenation^25^ at all levels of disease severity in adult patients^26^ with benefits upon the social and adverse health consequences of OSA and snoring (e.g., decreasing daytime sleepiness and improving quality of life)^25,26^.

Treatment of OSA with MAD improves both subjective and objective measures of excessive daytime sleepiness. Yet, the obtained subjective improvement may be also attributable to a placebo effect^34,35^.

A meta-analysis of seven randomized controlled studies confirms a modest, but rather significant benefit of MAD treatment on blood pressure^26^. The impact of treatment on other cardiovascular endpoints, such as cardiovascular events and mortality, remains unresolved^36^.

### MAD types

Since the first commercially available oral devices were introduced in the 1980s^36^ there was a proliferation in the development of several models of MAD but the lack of standardization for device design often hampered the interpretation regarding comparisons in clinical use and research results^22,25^. Table 1 characterizes the main types of FDA approved MAD for use in adult OSA treatment. MAD can be made of distinct materials and may have different designs, some of them with the ability of a progressive mandibular advancement and lateral movements. MAD are classified as prefabricated and customized^37-39^ in a single block (monoblock) or two blocks, which can be adjustable with some freedom in lateral movements^39^. MAD customization also involve material choice, which must be adapted to the oral structure and physical needs for each patient^38^.

**Table 1:** Some of the FDA-approved MAD that can be applied to improve mild to moderate obstructive sleep apnea in patients 18 years and older.

Technical data of the available MADs										
MAD	EMA	Narval	Pantera	NOA	Somnodent avant	Somnodent dorsal wings	Micro2	Herbst	Dream TAP	Orthoapnea classic
**Coupling mechanism**	Lateral superior-anterior to inferior-posterior	Lateral superior-anterior to inferior-posterior	Lateral superior-anterior to inferior-posterior	Lateral superior-posterior to inferior-posterior		Lateral superior-posterior to inferior-posterior	Lateral superior-posterior to inferior-posterior	Lateral superior-posterior to inferior-anterior	Anterior superior and inferior	Anterior superior and inferior
**Coupling element**	Lateral Straps	Lateral Straps	Lateral Straps	Cam/Follower	Anterior strap	Dorsal wings	Dorsal wings	Fixed rod	Anterior hook	Anterior rod
**Titration mechanism**	Diferent lenths of strips	Diferent lenths of strips	Diferent lenths of strips		Diferent lenths of anterior strip	Screw on lateral wings	Different lower splints	Screw on rod	Different lower splints	Screw on anterior rod
**Titration protocol**	"9 different lengths allow for 1 mm advancement of the mandible"	Different lengths	Different lengths	Customized titration	Initial set of 10 straps (+1mm) provided	One 360º turn of the screw is 0.4 mm / 6.0mm protrusion range	Customized titration	Range of advancement (8mm	One 360° turn of the srew is 0.5 mm / 15mm protrusion range	From-3mm to +7mm total range of screw 10mm
**Material**	Thermoformed splints	3D printed nylon appliance	3D printed nylon appliance	3D printed nylon appliance		Acrylic splint	Crystal-clear acrylic	Acrylic splint	Thermoformed splints	Thermoformed dual Laminate Splints
**Fabrication**	Tradicional laboratories	CAD/CAM-printed 3D	CAD/CAM-printed 3D	CAD/CAM-printed 3D	CAD/CAM-printed 3D	Tradicional laboratories	CAD/CAM-milled	Tradicional laboratories	Tradicional laboratories	Tradicional laboratories

### Prefabricated MAD

**Figure 1:**
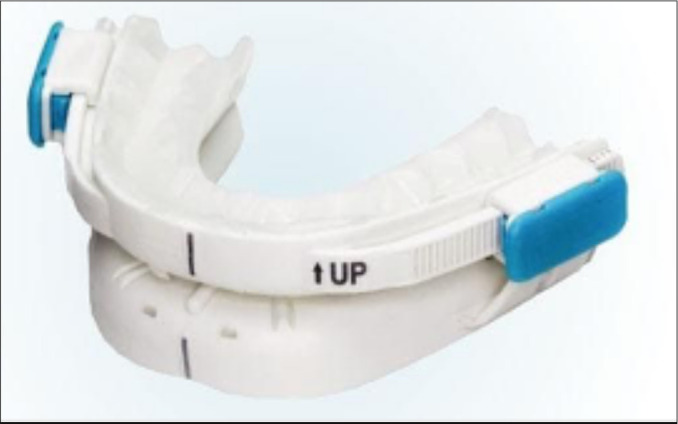
Prefabricated Intraoral mandibular advancement devices (MAD); titratable thermoplastic MAD BluePro® (France).

Prefabricated MAD tend to be bulky with some challenges regarding its retaining capacity on a stable mandibular protrusive position during sleep. Therefore, this type of MAD are more prone to loss efficacy and lead to patient’s discomfort^40,41^. Nevertheless, a recent study demonstrated the efficacy of a titratable thermoplastic MAD in reducing OSA and related symptoms in patients with mild to severe disease presentation, but more studies are still needed to evaluate their efficacy in OSA treatment^42^ (Figure 1).

### Custom-made MAD

The use of a custom-made MAD (MADc) has been associated with increased patient-reported comfort, greater range of protrusive movement, and higher therapeutic effectiveness^25,26^.

**Figure 2:**
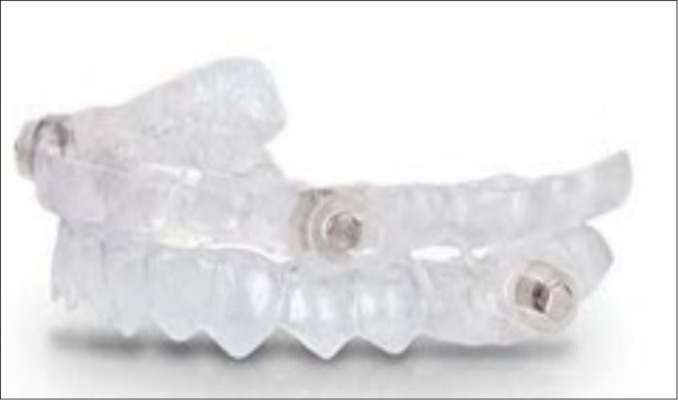
Intraoral mandibular advancement devices (MAD); a customized MAD‘s DIA Apnia®, Spain.

**Figure 3:**
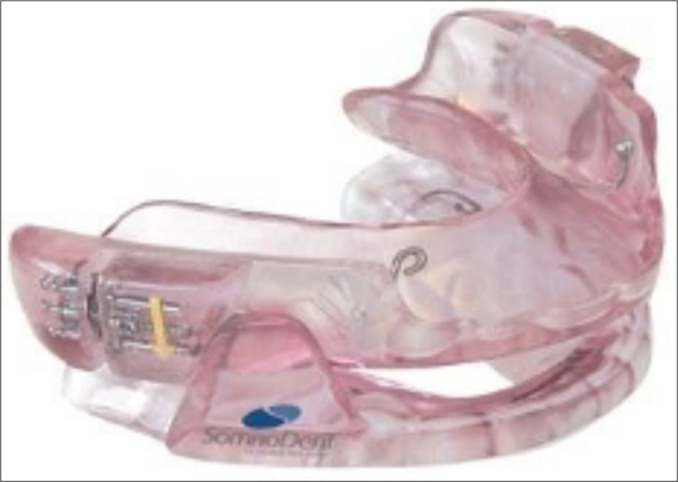
Intraoral mandibular advancement devices (MAD)a SomnoDent® (France)^42^.

**Figure 4:**
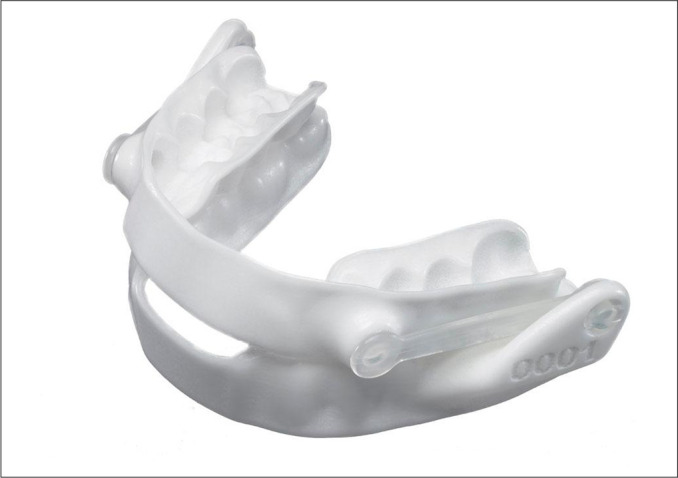
Intra oral mandibular advancement device (MAD) Narval CC, France.

**Figure 5:**
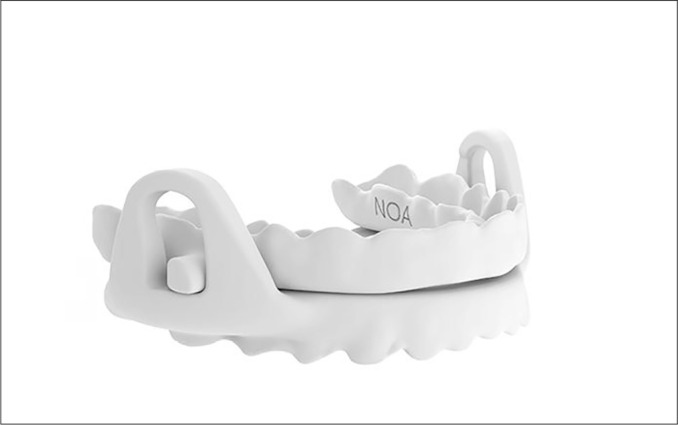
Intra oral mandibular advancement device (MAD) NOA, Spain.

MADc are tailored to the patient’s dentition in a laboratory-controlled advancement. This kind of appliance can be adjustable (bi-block) or non adjustable (monoblock). Clinically, non-adjustable MAD are made in a fixed protrusive position remaining unchanged during treatment, while the protrusive position of the adjustable MAD allows progressive advances (e.g, titration process) aiming to increase the treatment efficacy and the patient’s comfort and quality of life (Figures 2 to 5)^26,41,43^.

### Coupling mechanism

In the design of a non-adjustable MAD there are a variety of coupling mechanisms such as elastic straps, lateral fins, bars, telescopic rods, springs, and tube connectors among others^44^. The differences in the designs affect effectiveness and comfort. Devices with lateral coupling mechanism offer greater comfort than other systems because they allow mandibular lateral and protrusive movement while maximizing the space available for the tongue. Yet, the coupling mechanisms that allow a certain degree of opening and prevent the jaw from moving backwards at any time while opening the mouth are more effective^45-47^. Mouth opening can be limited by the integration of elastic bands in the design.

Although the range of protrusive movement differ between individuals, studies indicates that the ability to advance the jaw is a key feature in MAD treatment success. This mostly depends on the amount of progression needed to reduce apnea-hypopnea index (AHI) and snoring^38,40,43,48,49^. Nevertheless, a precise linear relationship between mandibular advancement and treatment success has not yet been shown^50^.

The mandibular protrusion mechanism must allow advancement in increments of 1mm or less, with a minimal range of 5mm^38,49^. Small increments allow the assessment of important parameters, pursuing to minimize, for example, the temporomandibular discomfort and to improve comfort^48,49^. Current data support an initial position of, at least, 50% of the patient’s protrusive range, but there is no consensus as to whether this should be measured from an initial position of maximum intercuspation or maximum retrusive mandibular position^49,51^. The mechanism should be reversible in order to adapt to the patient’s health related dynamics and to adequately manage side effects^48^.

Vertical adjustment has been another controversial subject in the design of oral appliances^25^. Although with a lack of consensus, some studies have suggested that increased vertical dimension (inter-incisal distance) resulted in reduced patient acceptance^38,52^. Indeed, an increase in inter-incisal distance during mandibular protrusion with MAD was shown to result in a slightly more retrusive position caused by posterior rotation of the mandible. This finding lead to the standard of avoiding vertical increase as much as possible^53^.

### MAD indications and effectiveness

MAD are indicated for patients with mild to moderate OSA and primary snoring. However, MAD are also an accepted therapy for patients with severe OSA who do not respond to or do not tolerate positive airway pressure (PAP) therapies^24,26^. Snoring treatment should be recommended for patients who fail to take conservative measures (e.g., weight loss, positional therapy, and avoidance of alcohol) and request additional treatment taking into account the risk/benefit ratio of the therapeutic action in view of the individual condition^26^. Contraindications such as periodontal damage and the presence of caries or poor oral hygiene should be prior evaluated by the dentist who should take care of the required dental treatments before MAD insertion. The risk of cclusal changes or exacerbation of temporomandibular joint disorders should also be considered and discussed with the patient^29^.

Although MAD are typically used as a single therapy, they can also be combined with CPAP and/or other therapeutic modalities for better OSA control. This is a relevant issue since patients report higher preference and higher adherence rates to treatment with MAD compared with CPAP devices^24,25,28^.

According to the American Academy of Sleep Medicine, one of the primary indications for MAD treatment is persistent snoring^26^. Such treatment indication occurs without the presence of a standardized classification in terms of severity or frequency of primary snoring, despite studies showing its possible relationship with factors associated with cardio-metabolic risk (e.g., atherosclerosis and endothelial dysfunction)^54-56^. Numerous questions related to this topic still remain unanswered. For example, questions like when treatment is needed and in which situations snoring is considered to be harmful are still controversial and would benefit of further debate^54^.

MAD have been shown to improve polysomnographic based outcomes, reduce AHI, arousals and the rate of oxygen desaturation^26^, also with significant improvements in daily function and quality of life. However, treatment with MAD failed to demonstrate a significant effect on sleep architecture and efficiency. Despite being less effective than CPAP in AHI improvement in moderate to severe OSA, several recent studies have found that oral appliances and CPAP were almost equally effective in tackling daytime sleepiness, hypertension, neurocognitive function, quality of life, and cardiovascular mortality^25^. A study from Hoekema et al. (2008)^57^ compared polysomnographic data between MAD and CPAP at 8-12 weeks with MAD being adjusted until the AHI<5 was obtained or until they caused discomfort. Results showed that 76.5% of the MAD patients’ group was effectively treated (with 69.2% critically ill patients being considered effectively treated and 84.0% of non-severe patients were considered effectively treated) versus 82.7% of the CPAP group^57^.

Although MAD are more effective than other types of OA in OSA treatment, their available design features (e.g., used materials, method of manufacture, and device adjustability) together with the disease severity might have a different impact on treatment effectiveness^38,58,59^. In fact, several studies have reported that for a higher efficacy, an advancement greater than 50% of a patient’s maximum protrusion will be required^48,51,57,60,61^.

Anatomical features resulting in increased nasal resistance seem to have a negative impact in the treatment with MAD^17^. Hence, studies evaluating the importance of nasal breathing in candidates for MAD found that those who had increased nasal resistance responded less effectively and had less adherence to treatment when compared to patients without nasal changes^62^.

Studies have also tried to prove the efficacy of treatment with MAD by imaging tests. Convincing evidence of improvement in airway permeability with MAD use was obtained by drug-induced sedation endoscopy (DISE), an objective method for visualizing upper airway obstruction^25,63^.

Numerous imaging studies have been performed in awake patients to elucidate tissue changes after mandibular advancement that could predict improved breathing during sleep. The use of computed tomography (CT-scan) and magnetic resonance imaging (MRI) in awake patients revealed that the greatest changes in the airways after MAD insertion were frequently observed in the transversal dimension, limiting the interpretation of the two-dimensional cephalometric images of the sagittal plane, and therefore failing to provide a reliable way of predicting treatment results^25^. Three-dimensional examinations at wakefulness and sleep conditions with and without MAD revealed that MAD increase the retropalatal and retro-lingual spaces and decrease the length of the soft palate. The increase in the retropalatal space is a crucial indicator for a positive treatment outcome^64,65^.

### Treatment adherence and follow-up

In general, studies show that adherence to MAD is higher when compared to CPAP in adult patients with OSA. Indeed, the larger adherence to MAD seems to compensate for its lower efficacy in relation to CPAP, resulting in significant improvements in most clinical and polysomnographic outcomes, the so-called average disease relief factors^66^. However, we must consider that, unlike CPAP, there is little objective data to assess adherence to MAD^26^. In an interesting study, Vanderveken et al. (2013)^28^ showed an average use of MAD per night of ~6.6h in 82% of the evaluated patients.

Among the distinct MAD available types, the most effective one meets both the therapeutic success criteria and the higher patient acceptance. This highlights the role of a trained dentist in the treatment of OSA and the need of a personalized treatment using MAD.

Due to its lifelong nature, MAD treatment for OSA requires a careful and prolonged monitoring of patients through sleep tests to confirm treatment effectiveness as subjective feedback is not sufficient to follow-up MAD therapeutic effects in OSA management^67^. In fact, without objective data, the patient may remain suboptimally treated. Follow-up of adherence, MAD deterioration/maladjustment, health of oral/craniofacial structures, and integrity of the occlusion should be performed. Sleep monitoring test might also be considered for the search of worsening OSA signs and in treated patients who develop recurrent symptoms, present weight gain or receive diagnoses of comorbidities relevant to OSA.

### MAD therapy side-effects

The side effects resulting from MAD treatment are of short- and long-term nature. Short-term effects are generally mild, transient, and occur within 6 months of MAD application. Such effects, usually controllable by a sleep-trained dental professional, include excessive salivation, dry mouth, discomfort in the teeth, irritation in the gums, headaches, and discomfort in the temporomandibular joint and in the masticatory muscles^68^. The long-term side effects occurring beyond the 6 months after the initiation of treatment have a poor prognosis and are most often related occlusal changes^67,69^. Among these effects are the decreased overbite and overjet, lingual inclination of the upper incisors, the vestibular inclination of the lower incisors, the mesialization of the lower molars and distalization of the upper molars, changes in dental arches crowding, appearance of posterior open bite, and decreased occlusal contacts^69-72^. Dental changes develop as a result of the MAD exerted forces on the upper and lower dental arches in order to maintain protrusion, and jaw resistant counter forces to persist in its initial position^72^. A 21-year follow-up study on the monitoring of MAD side-effects confirmed that there are significant and progressive dental changes with the prolonged use of MAD but skeletal or postural changes were insignificant. In addition, the duration of treatment was the most consistent predictor associated with the magnitude of the observed side effects^67^.

A qualified MAD provider should be able to individually assess each patient, choose the best MAD and adapt it in order to evaluate and minimize its side effects. Adequate evaluation in accordance with the individual’s health condition is critical, as some of the observed side effects such as destabilization of the occlusion may not revert^26,67,73,74^. In this context, patients should be aware of such probable effects before starting the treatment.

### Patients’ phenotypes and prediction of outcomes

There is a growing recognition that OSA is a heterogeneous disorder either in terms of risk factors, clinical presentation, pathophysiology, risk of comorbidity, or response to treatment. A better characterization of this heterogeneity is an essential step for personalized approaches to therapy, ensuring more effectiveness and adherence to the proposed treatment^75^.

Recent studies on patients’ phenotypes responding to MAD treatment have shown that about one third of the included patients have little or no reduction of OSA severity^76^. These data display the critical need of identifying which patients are most likely to have a positive treatment outcome eventually leading to an optimization regarding the choice of the treatment modality, search for adjuvant measures and also to avoid wasting medical resources^77^. Drug Induced Sleep Endoscopy DISE titration can be used as a predictor of MAD treatment outcomes due to a direct assessment of upper airways obstruction sites as well as a way to prospectively determine which patients will have the best response to treatment and which mandibular protrusion should be targeted^78-80^. Recently, anatomical and physiological phenotypes have been identified as critical variables for the choice and the prediction of OSA treatment success and they include both anatomical and functional factors such as critical pressure to close the upper airways (Pcrit), arousal threshold and muscle response capacity, all of them playing an important role in OSA pathophysiology^81-87^. OSA-related additional comorbidities such as hypertension and/or other sleep disturbances (e.g., insomnia) can negatively influence the response to MAD treatment^83,88^.

Nevertheless, updated knowledge on the extent to which these factors interfere in OSA treatment with MAD is still needed.

### Phenotypic characteristics prone to MAD treatment response (level of evidence)

**Table 2:** Major phenotypic characteristics prone to MAD treatment according to levels of evidence.

Clinical characteristics	Lower age, female gender, lower BMI, lower neck circumference (strong)
Craniofacial profile	Mandibular an maxillary retrognathism, smaller airway, shorter soft palate (strong)
PSG parameters	Mild OSA (moderate) (strong), Positional OSA
Physiological parameters	Primary oropharyngeal collapse, low CPAP therapeutic pressure (weak)

Adapted from Chen et al. (2020)^77^.

### Sleep dentist as a qualified MAD provider

There are currently many specializations and post graduated programs in sleep medicine directed towars dentists. Such an achievement is important for screening, diagnosis, optimized clinical approaches, and technical adequacy of therapeutic tools provided by dental professionals. Although the dentist’s role in sleep medicine should depend on their specific knowledge and expertise, sleep apnea is a core issue in any dental sleep medicine program^89^. It is well accepted that MAD treatment require qualified knowledge and expertise from a dentist trained in sleep medicine, therefore allowing for an optimal therapeutic achievement particularly related to proper MAD selection and treatment decision algorithms aiming to prevent occlusal and pain related issues as well as adequate referral when indicated Standards of care were provided by American Academy of Dental Sleep Medicine for this purpose^90^. These standards will be probably associated with higher rates of success as well as lower rates of complications and/or adverse issues.

## Conclusions and future perspectives

Current literature provides robust evidence that adjustable and personalized double-arch oral mandibular advancement devices are highly effective for the treatment of snoring and mild to moderate OSA. Although less effective than CPAP for improving AHI in moderate to severe OSA, several recent studies have found that oral appliances and CPAP were equally effective in improving daytime sleepiness, hypertension, neurocognitive function, quality of life, and cardiovascular mortality. Further studies are needed to establish the impact of different models of devices available on therapeutic success and patient compliance as well as related side effects.

A qualified MAD provider needs to have the necessary skills to choose, adjust and manage the side effects of the most appropriate device. MAD treatment protocol should only start after a medical evaluation based on standard clinical, physical and polysomnographic parameters. A careful candidate selection must be carried out by both the sleep specialist and qualified MAD provider in order to achieve a higher therapeutic success rate. Still, the development of tools to identify individual phenotypes and the combination of two or more therapies to obtain synergistic additive effects will be required for the adoption of an optimal personalized OSA treatment.
